# Experimental study on the effects of blending PODE_n_ on performance, combustion and emission characteristics of heavy-duty diesel engines meeting China VI emission standard

**DOI:** 10.1038/s41598-021-89057-y

**Published:** 2021-05-04

**Authors:** Yuwei Zhao, Cong Geng, Weibo E, Xiaoquan Li, Peiyuan Cheng, Tianlin Niu

**Affiliations:** 1 Air and Missile Defense College, Air Force Engineering University, Xi’an, 710051 People’s Republic of China; 2 College of Foreign Studies, Guilin University of Technology, Guilin, 541004 People’s Republic of China

**Keywords:** Fossil fuels, Renewable energy

## Abstract

To study the influence of diesel fuel blended with polyoxymethylene dimethyl ethers (PODE_n_), a new alternative fuel with a high oxygen content and large cetane number, on the combustion characteristics, fuel economies, and emission characteristics of heavy-duty diesel engines that meet China VI emission standards, relevant tests were conducted on a supercharged intercooled high-pressure common-rail diesel engine. The PODE_n_ were blended with diesel fuel at three different ratios (volume fractions of 10%, 20%, and 30%). The test results showed that the PODE_n_ could optimize the combustion process of diesel engines that met the China VI emission standards, effectively improve the thermal efficiencies of diesel engines, and reduce the emissions of hydrocarbon (HC), carbon monoxide (CO), and soot. With an increase in the PODE_n_ blending ratio, the peak values of the in-cylinder pressure, average in-cylinder temperature, and instantaneous heat release rate gradually decreased, and each peak progressively moved forward. As the start of combustion gradually moved forward, the combustion duration was shortened by 0.7–2.8°CA, the heat release process became more concentrated, and the effective thermal efficiency was increased by up to 2.57%. The effective fuel consumption gradually increased, yet the equivalent effective fuel consumption gradually decreased, with the largest drop being as high as 4.55%. The nitrogen oxides (NO_*x*_) emission increased slightly, and the emissions of HC, CO, and soot gradually decreased. The emissions of CO and soot declined significantly under high-speed and high-load conditions, with the highest reductions reaching 66.2% and 76.3%, respectively.

## Introduction

To address the environmental pollution and energy crises prevailing worldwide, countries around the world continue to implement increasingly stringent regulations related to the discharge of pollutants. The EU began to implement European VI, VI b, and VI c emission standards in January 2013, September 2014, and September 2017, respectively. Compared with the European V emission standard, the European VI emission standard contained added emission limits for the particle number (PN), and the on board diagnostics (OBD) limit was even more stringent. Proposed by the Environmental Protection Agency (EPA) in March 2013, the Tier 3 fuel and vehicle standards will be implemented in phases from 2017 to 2025. The EPA’s Tier 3 limits the total emissions of “NMOG (nonmethane organic gas) + NO_*x*_.” China plans to fully implement the China VI a emission standards in 2020 and the China VI b emission standards in 2023. Shenzhen took the lead in implementing the China VI a emission standard at the end of 2018, while Shanghai, Guangzhou, Tianjin, and other cities began to enforce the China VI b emission standard on July 1, 2019. Formulated based on both the European VI c and EPA Tier 3 emission standards in terms of the requirements for the OBD and the evaporative emissions, the China VI emission standard is known as the strictest emission standard in the world.

Diesel engines are widely used in transportation, agriculture, and industry^1–3^. Facing increasingly strict emissions regulations and a gradually growing energy crisis, investigators are pursuing the efficient and clean combustion of diesel. The optimization of fuel characteristics by blending oxygenated fuels is an effective way to achieve this objective^4–6^. Once the oxygenated fuel is blended with diesel, the physical and chemical properties of the blended fuel, e.g., the oxygen content, cetane number, and latent heat of evaporation, are changed accordingly, thereby altering the spray characteristics, combustion features, power performance, and emission characteristics of diesel engines^7–11^. Alcohols (e.g., methanol^12^ and ethanol^13,14^), ethers (e.g., dimethyl ether^15^), and esters (e.g., biodiesel^16^) are currently the primary oxygenated fuels blended in diesel. Oxygenated fuels such as alcohols, ethers, and esters are strongly competitive for improving the combustion process and pollutant emissions of diesel engines. However, significantly different from diesel in terms of overall physical and chemical properties, these fuels are restricted in various ways in practical applications^17,18^. Therefore, it is of great practical significance to find oxygenated fuels with similar physical and chemical properties to those of diesel.

As a new type of oxygenated additive for diesel, polyoxymethylene dimethyl ethers (PODE_n_) are chemically expressed as CH_3_O(CH_2_O)_n_CH_3_ (the degree of polymerization n is an integer, 2 ≤ n ≤ 8). With physical and chemical properties fairly similar to those of diesel, PODE_n_ are liquid at 20 °C and they can be miscible with diesel in any proportion. With a high oxygen content (45.2–51%), a large cetane number (above 63), and without a C–C bond in their molecular structure, PODE_n_ are able to effectively reduce the soot emissions during the combustion process of diesel engines^19–22^. In addition, the production costs of PODE_n_ are close to those of diesel, and with the advancement of production technology and the expanded production scale of PODE_n_, the production costs of PODE_n_ are expected to be further reduced, which makes it possible for PODE_n_ to be applied to diesel engines on a large scale. Hence, PODE_n_ are a type of clean alternative fuel that exhibit excellent performances and great potential^23–30^.

Recently, a number of studies have been carried out to study the effects of blending PODE_n_ into diesel on diesel engines with different emission standards. By fueling a light-duty diesel vehicle with the Euro II emission standard using diesel blended with 10% PODE_n_, Pellegrini et al.^31^ reduced the particulate matter (PM) emissions by 18%, while if pure PODE_n_ were used, the PM emissions could be decreased by 77%, meeting the Euro IV emission standard. By blending 7.5% PODE_n_ into the diesel used on a Euro III light-duty diesel engine, Pellegrini et al.^32^ found that compared with pure diesel, diesel blended with PODE_n_ reduced the PM emissions and the exhaust smoke by 13% and 32%, respectively, with increased emissions of polycyclic aromatic hydrocarbons (PAHs) and PM with particle sizes smaller than 30 nm. Liu et al.^33^ burned diesel blended with 15% and 25% PODE_n_ in a Euro IV diesel engine, indicating that blending PODE_n_ could significantly improve the trade-off relationship between the emissions of NO_*x*_ and the soot of the diesel engine. Under the WHSC test cycle, with the NO_*x*_ emissions controlled at 2.7 g/(kW·h), the soot emissions met the Euro VI emission standard. Liu et al.^34^ studied the effect of the PODE_n_ blending ratio on the performance of a diesel engine with China IV emission standards, and the results showed that as the PODE_n_ blending ratio increased, the ignition delay period shortened, the in-cylinder temperature gradually increased, the soot emission was reduced by up to 47.6%, and the effective thermal efficiency could be increased by up to 3.4%. Wang et al.^35,36^ explored the impact of blending PODE_n_ on the combustion and emission characteristics of light-duty and heavy-duty diesel engines, and the results indicated that blending PODE_n_ prolonged the ignition delay period and increased the peak heat release of the main injection. When 20% PODE_n_ were blended, the soot and CO emissions of a light-duty diesel engine could be reduced by 90%, the PM emissions of a heavy-duty diesel engine during the ESC (European Stationary Cycle) test cycle were reduced by 36.2%, and the effective thermal efficiency was increased by 0.85%. Moreover, to further improve the physical and chemical properties of fuels, Huang et al.^37^ and Chen et al.^38^ studied a ternary fuel mixture of diesel, PODE_n_, and high-octane fuel, and the results indicated that blending a certain proportion of PODE_n_ in the diesel or high-octane fuel could reduce the emissions of NO_*x*_ and soot.

Therefore, blending PODE_n_ can effectively improve the combustion and emission characteristics of diesel engines, especially when it comes to reducing the emissions of soot and PM. It also has been shown that the same fuel has different combustion and emission performances in different types of diesel engines^39^. Likewise, there are certain differences in the effects of blending the same proportion of PODE_n_ into the diesel fuel on the combustion characteristics of different types or models of engines as well as the effects on the emissions of NO_*x*_, soot, and CO^26,31,34,36,40,41^. Many studies have confirmed the feasibility of adding PODE_n_ to diesel engines, yet there are few experimental studies regarding PODE_n_ blending with the diesel used in diesel engines that meet the China VI emission standards. Given that China is about to fully implement the China VI emission standards, it is necessary to carry out such studies on PODE_n_/diesel blended fuels with China VI diesel engines. To this end, with a supercharged, intercooled, high-pressure common-rail diesel engine with the China VI emission standards as the research subject, the influences of the PODE_n_ blending ratio on the combustion and emission characteristics as well as the fuel economy of a heavy-duty diesel engine were investigated in this study. The results will provide empirical support for optimizing the fuel characteristics and combustion process of PODE_n_ as an alternative fuel for China VI emission standards and the revision of the release control and post-treatment strategies.

## Materials and methods

### Testing apparatus

A Weichai Power WP12.460 China VI heavy-duty diesel engine, an in-line 6-cylinder, supercharged, intercooled, electronically controlled high-pressure common-rail diesel engine was used in the tests. The technical specifications of the engine are shown in Table 1.

**Table 1:** Technical specifications of the diesel engine.

Parameter	Specifications
Engine type	In-line 6-cylinder, 4-stroke, supercharged, intercooled, high-pressure common-rail
Bore × stroke (mm × mm)	126 × 155
Compression ratio	17
Displacement (L)	11.596
Rated power (kW)	338
Rated speed (r·min^−1^)	1900
Fuel injection system	Bosch CRSN2-16

**Figure 1:**
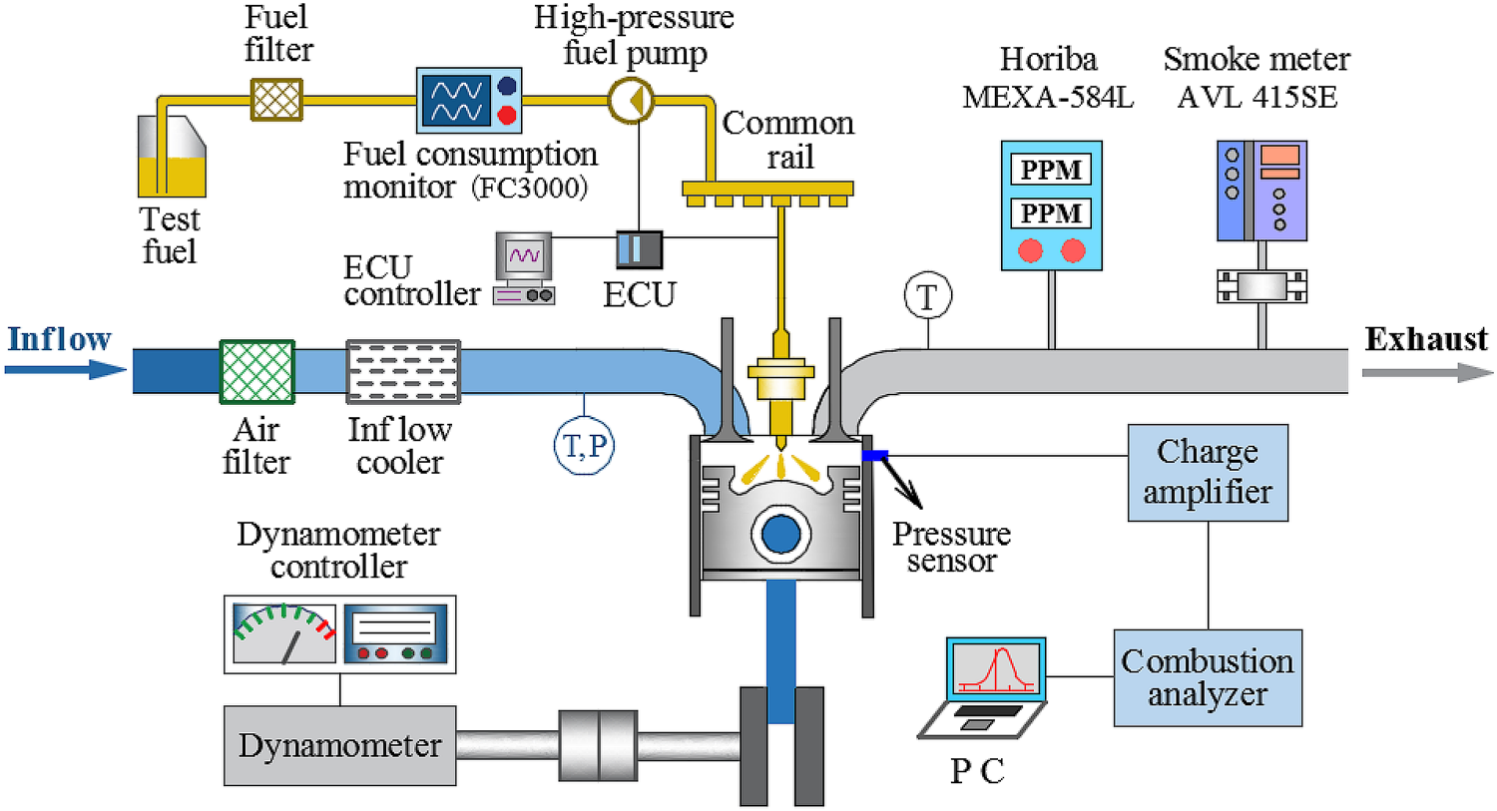
Schematic diagram of the testing apparatus.

The testing apparatus was mainly composed of an electric eddy current dynamometer, a combustion analyzer, a mass flow meter, a coolant and oil thermostatic system, and an exhaust gas analyzer. Figure 1 shows the schematic layout of the testing apparatus. The Kistler6052C cylinder pressure sensor was used to collect the in-cylinder pressure, which was amplified and recorded by the Kistler5064 charge amplifier built inside the KiBox combustion analyzer. The heat release rate and the other combustion characteristics data were calculated by the KiBox combustion analyzer. The Horiba MEXA-584L exhaust gas analyzer was used to measure the emissions of NO_*x*_, HC, and CO, and the AVL 415SE smoke meter was used to measure the exhaust soot. The Siemens FC3000 mass flow meter was used to measure the fuel consumption.

### Testing fuels

The PODE_n_ used in the study were industrial PODE_n_ with a purity of 99.9% that were produced by Shandong Yuhuang Chemical (Group) Co., Ltd., and they were primarily composed of PODE_2_, PODE_3_, and PODE_4_ with mass fractions of 2.553%, 88.9%, and 8.48%, respectively. The test diesel was 0^#^ commercial diesel. In this test, three kinds of PODE_n_/diesel blended fuels were prepared, with PODE_n_ volume percentages of 10%, 20%, and 30%, which were denoted as P10D90, P20D80, and P30D70, respectively, and the pure diesel was denoted as P0D100. The physical and chemical properties of the fuel are shown in Table 2. The physical and chemical properties of P10D90, P20D80, and P30D70 were calculated based on Eqs. (1) and (2) from a previous study^42^.

**Table 2:** Physical and chemical parameters of the fuels.

Properties	Diesel	PODE_n_	P10D90	P20D80	P30D70
Cetane number	56.5	78.6	58.71	60.92	63.13
Low heat value (MJ·kg^−1^)	42.5	19.05	39.76	37.11	34.57
Oxygen mass fraction (%)	0	46.98	5.49	10.79	15.89
Density at 25 °C (g·cm^−3^)	0.855	1.02	0.872	0.888	0.905
Kinematic viscosity at 25 °C (cSt)	3.44	1.05	3.16	2.89	2.63
Boiling point (°C)	188	158.49	184.55	181.22	178.02

### Testing protocol

Four different loads under two speeds, i.e., n = 1300 and 2000 r·min^−1^, were tested, with brake mean effective pressure (BMEP) values of 0.38 MPa, 0.76 MPa, 1.14 MPa, and 1.52 MPa. During the test, except for the fuel blend, the fuel injection strategy and the other control parameters remained constant, and the exhaust gas recirculation (EGR) valve remained closed. Under different operating conditions, the fuel injection strategy was the two-stage injection of the original machine: “pre-injection + main injection,” and the injection time and pressure were based on those of the original machine. Both the cooling water temperature and the oil temperature were controlled at about 80 °C. The ambient temperature for the test was 22 °C ± 3 °C, and the atmospheric humidity was 10% ± 3%.

Prior to the tests, all the instruments and the equipment were calibrated to ensure the reliability of the test and the accuracy of the test results. During the test, each operating point was repeatedly measured to eliminate uncertainty. After the engine was operating stably for 5 min, the fuel consumption was measured three times, and the average value was taken as the test result. The combustion analyzer collected the combustion characteristics data for 200 cycles and the average value of the data was taken for analysis. The exhaust gas analyzer and the smoke meter continued to be measured for 1 min, and with the invalid values eliminated, the average values were taken as the test results.

## Results and discussion

### Effects of blending PODE_n_ on combustion characteristics of diesel engines

**Figure 2:**
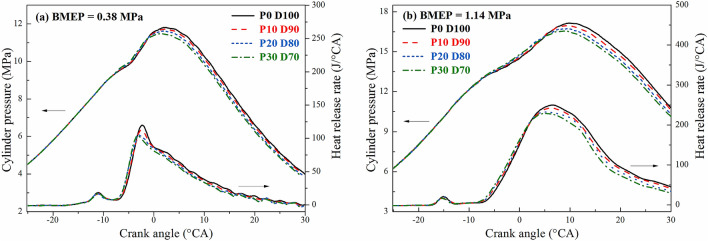
Effects of PODE_n_ blending ratio on in-cylinder pressure and instantaneous heat release rate.

Figure 2 shows the variation of the in-cylinder pressure and instantaneous heat release rate of the diesel engine with different PODE_n_ blending ratios when n = 2000 r·min^−1^, BMEP = 0.38 MPa, and BMEP = 1.14 MPa. Since the fuel injection consisted of two stages, the exothermic process was divided into two phases: pre-injection and main-injection heat release. Since the pre-injection lasted for a relatively short time, the amounts of fuel injection and heat released were small, and the blending of the PODE_n_ exerted no significant effect on the peak value of the pre-injection heat release rate. However, since the duration of the main-injection phase was longer, the peak value of the main-injection heat release rate decreased with the increase in the PODE_n_ blending ratio, with the peak phase gradually moving forward. Because the PODE_n_ had a lower heat value than diesel, under the injection conditions controlled by the electronic control unit (ECU) of the original engine, the blended fuel emitted less heat than the diesel with a declining peak value of the heat release rate. Moreover, the PODE_n_ had a large cetane number, which shortened the ignition delay period of the blended fuel and advanced the ignition timing.

Figure 2 also shows that as the PODE_n_ blending ratio increased, the maximum in-cylinder pressure decreased, with the corresponding phase of the peak in-cylinder pressure gradually moving forward. Due to the large cetane number, great self-ignitability, and good volatility of the PODE_n_, the ignition delay period of the blended fuel became shorter, with the corresponding phase of the peak in-cylinder pressure moving forward. The peak in-cylinder pressure decreased due to the following two factors. First, the lower heat value of the PODE_n_ reduced the heat value of the blended fuel and the heat release during the main-injection phase. Second, the shortening of the ignition delay period of the blended fuel caused the start of combustion (SOC) to occur earlier, shortened the mixing time of the fuel and the air, and reduced the premixed combustion ratio.

**Figure 3:**
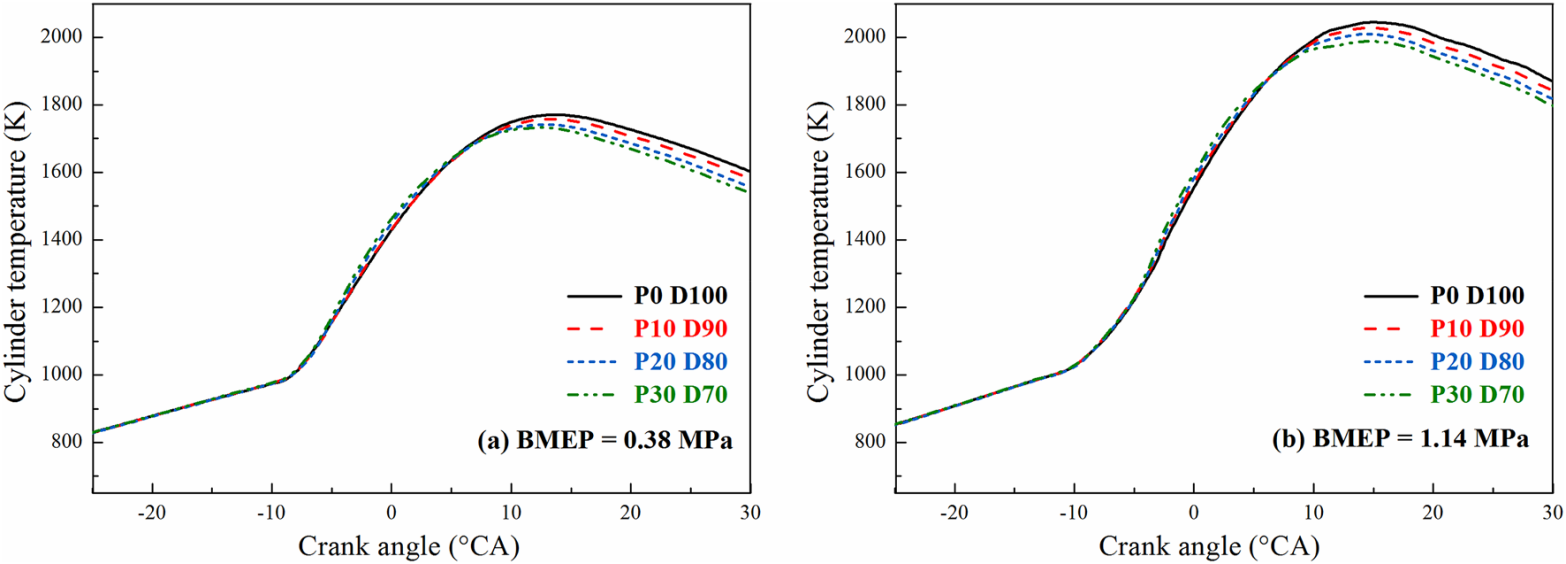
Effects of PODE_n_ blending ratio on in-cylinder temperature.

Figure 3 shows the variation of the average in-cylinder temperature with different PODE_n_ blending ratios when n = 2,000 r·min^−1^, BMEP = 0.38 MPa, and BMEP = 1.14 MPa. As the PODE_n_ blending ratio increased, the peak in-cylinder temperature dropped and the curve shifted forward. Since the latent heat of vaporization of the blended fuel increased after the blending of the PODE_n_, after the fuel was injected into the cylinder, part of the heat was absorbed, resulting in a relatively significant temperature drop, which led to a decrease in the peak in-cylinder temperature. However, due to the large cetane number of the PODE_n_, the combustion heat release of the blended fuel was advanced, thereby shifting the peak in-cylinder temperature forward.

**Figure 4:**
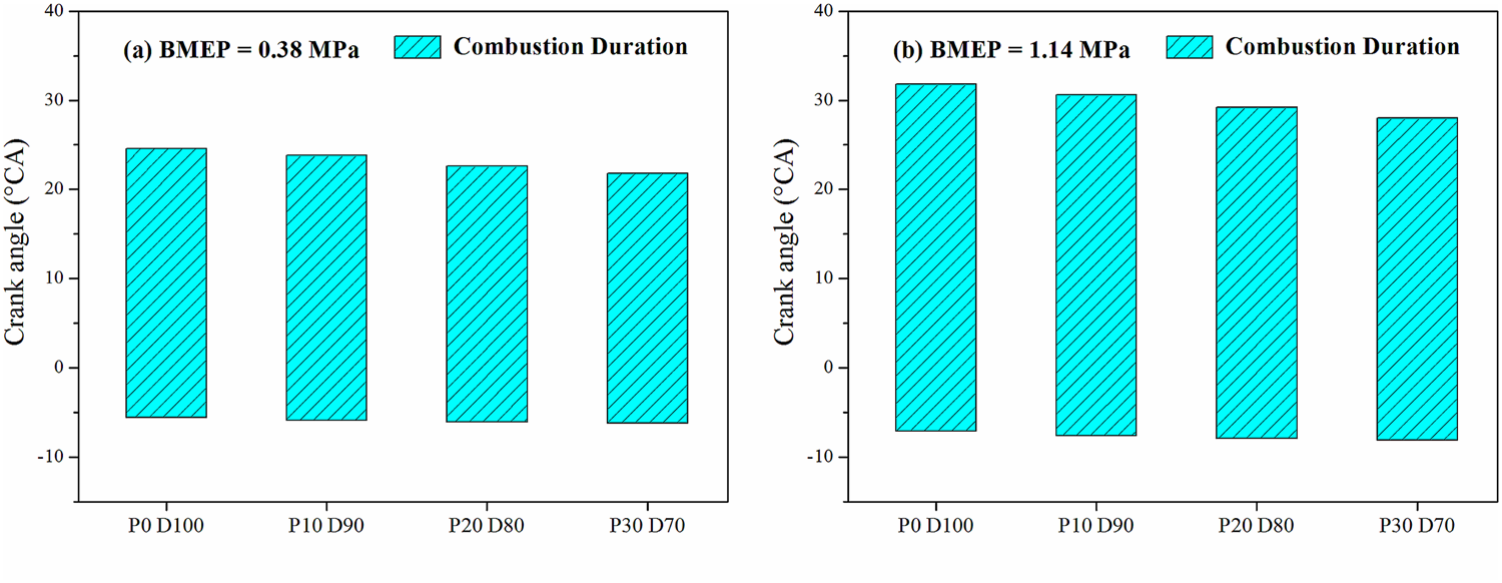
Effects of PODE_n_ blending ratio on combustion duration.

Figure 4 shows the variation of the combustion duration of the engine with different PODE_n_ blending ratios when n = 2,000 r·min^−1^, BMEP = 0.38 MPa, and BMEP = 1.14 MPa. In this study, the crank angle corresponding to a cumulative heat release rate of 10% (CA10) was defined as the SOC, the crank angle corresponding to a cumulative heat release rate of 90% (CA90) was defined as the end of combustion (EOC), and the crank angle between the SOC and the EOC was defined as the combustion duration. With BMEP = 1.14 MPa, compared with P0D100, when P10D90, P20D80, and P30D70 were used, the CA10 was advanced by 0.5°CA, 0.8°CA, and 1.0°CA, respectively, and the CA90 was advanced by 1.2°CA, 2.6°CA, and 3.8°CA, respectively, and the combustion duration was shortened by 0.7°CA, 1.8°CA, and 2.8°CA, respectively. This indicated that as the PODE_n_ blending ratio increased, the combustion duration was gradually shortened, and the heat release was more concentrated, which was conducive to improving the thermal efficiency of the engine.

**Figure 5:**
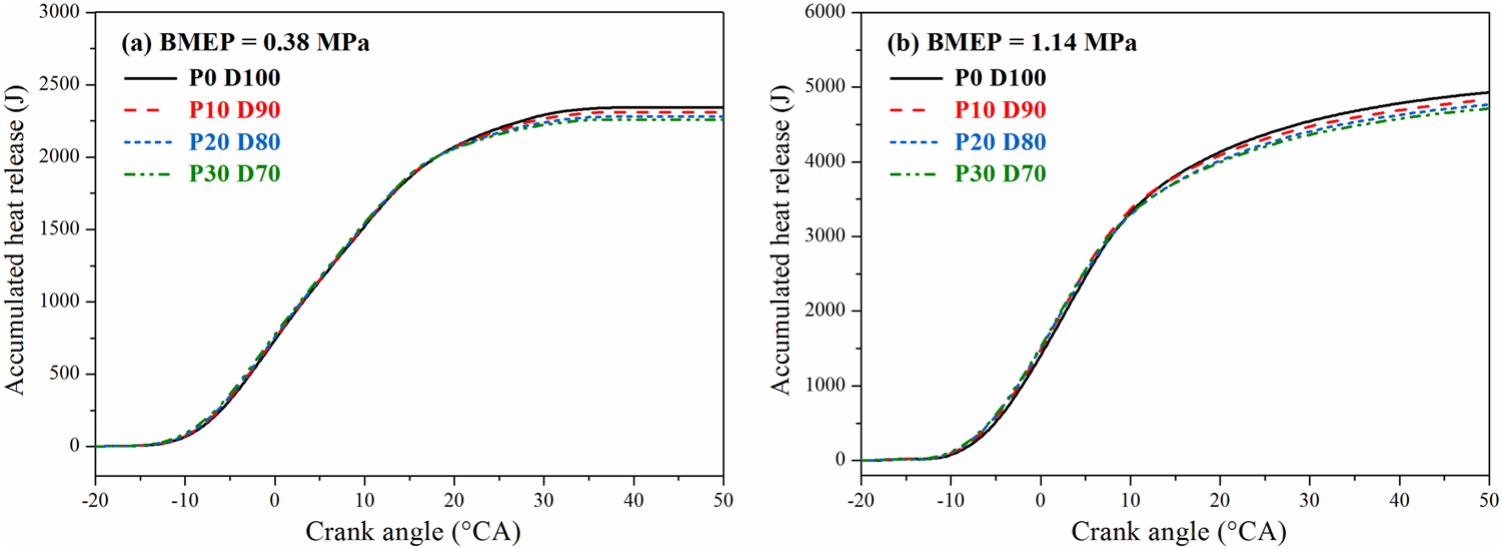
Effects of PODE_n_ blending ratio on cumulative heat release.

Figure 5 shows the variation of the cumulative heat release of the engine with different PODE_n_ blending ratios when n = 2,000 r·min^−1^, BMEP = 0.38 MPa, and BMEP = 1.14 MPa. The cumulative heat release decreased after the PODE_n_ were blended, and with the increase in the blending ratio, the cumulative heat dropped more significantly. At a BMEP of 1.14 MPa, when P0D100, P10D90, P20D80, and P30D70 were used, the maximum cumulative amounts of heat released were 4,959.9 J, 4,863.6 J, 4,789.1 J, and 4,733.6 J, respectively. Compared with P0D100, the cumulative amounts of heat released were reduced by 1.94%, 3.44%, and 4.56% when P10D90, P20D80, and P30D70 were burned, respectively. At the same speed and load, the effective power values of the engine were equal for all the blends. The lower the cumulative heat release was, the higher the thermal efficiency of the engine was. This indicated that blending PODE_n_ helped to improve the effective thermal efficiency of the engine. This conclusion is further discussed in “Effects of blending PODE_n_ on fuel economy of diesel engines”.

### Effects of blending PODE_n_ on fuel economy of diesel engines

The effective fuel consumption *b*_*e*_ and the effective thermal efficiency *η*_*et*_ are two important parameters that characterize the fuel economy of the engine, and they are critical indicators for measuring the engine performance. Because the low heat value of PODE_n_ differs greatly from that of diesel, directly comparing the fuel consumption of the fuels could not accurately reflect the total heat consumed by the fuels. Therefore, the concept of equivalent effective fuel consumption *b*_*eq*_ was introduced; i.e., the total heat value consumed by the PODE_n_/diesel blended fuel was converted to the diesel consumption corresponding to an equivalent heat value.

**Figure 6:**
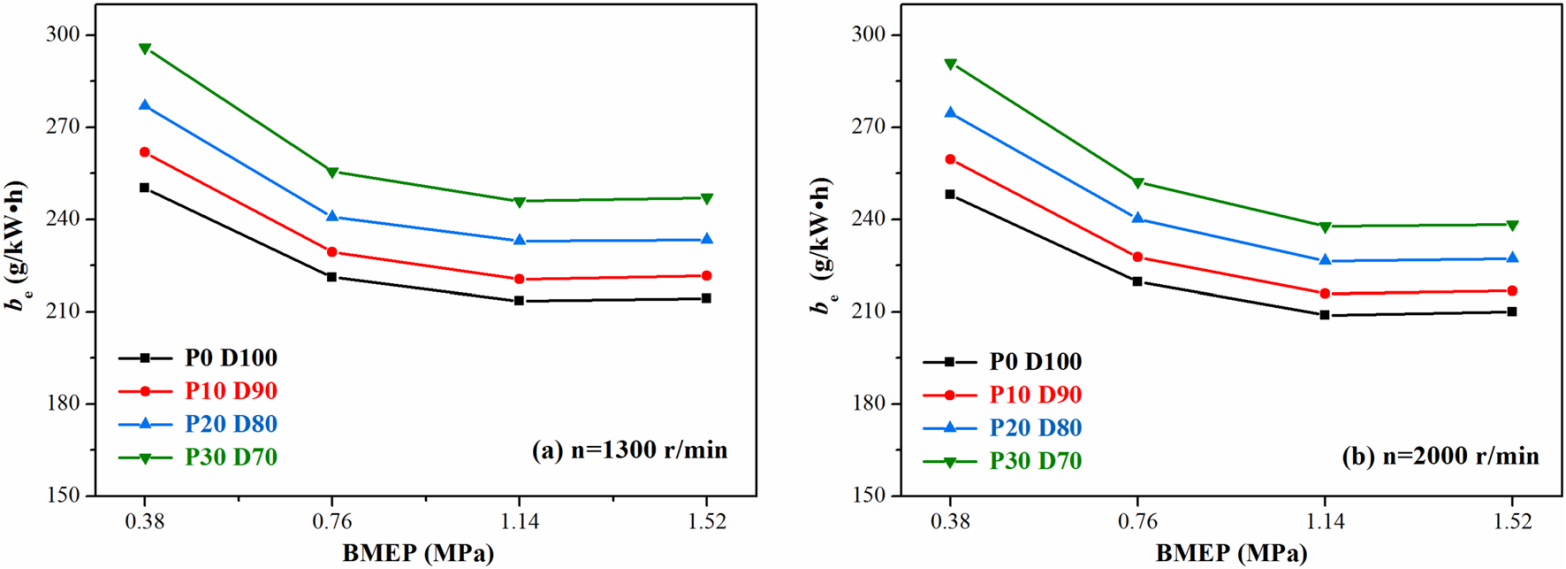
Effects of PODE_n_ blending ratio on effective fuel consumption.

The variations of the effective fuel consumption *b*_*e*_ with different PODE_n_ blending ratios are shown in Fig. 6. Given the same speed and load, the effective fuel consumption gradually increased with the increase in the PODE_n_ blending ratio. Due to the low heat value of the PODE_n_, the heat values of the blended fuels P10D90, P20D80, and P30D70 were 93.6%, 87.3%, and 81.3% of that of diesel, respectively, and the amount of heat released by combustion within the unit mass of the blended fuel was reduced. Without changing the fuel injection strategy, the output torques corresponding to the same BMEP operating condition were the same. Hence, it was important to increase the injection amount of the blended fuel, resulting in an increase in the effective fuel consumption.

**Figure 7:**
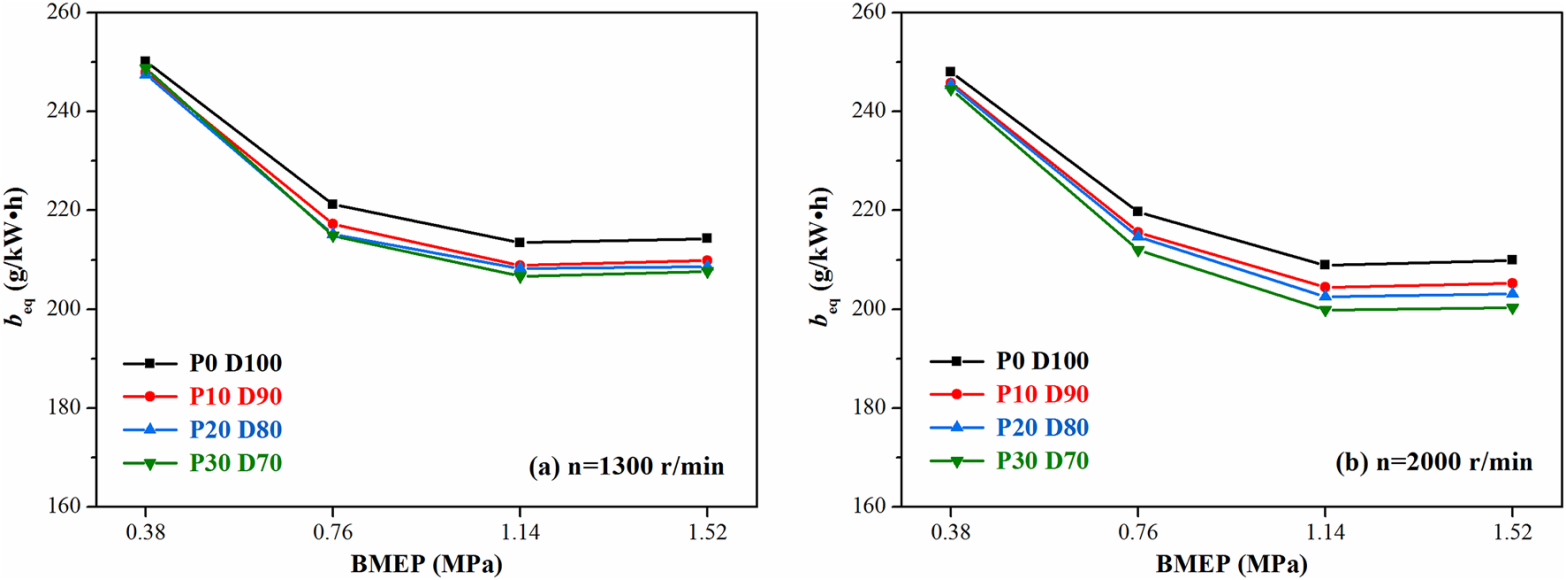
Effects of PODE_n_ blending ratio on equivalent effective fuel consumption.

**Figure 8:**
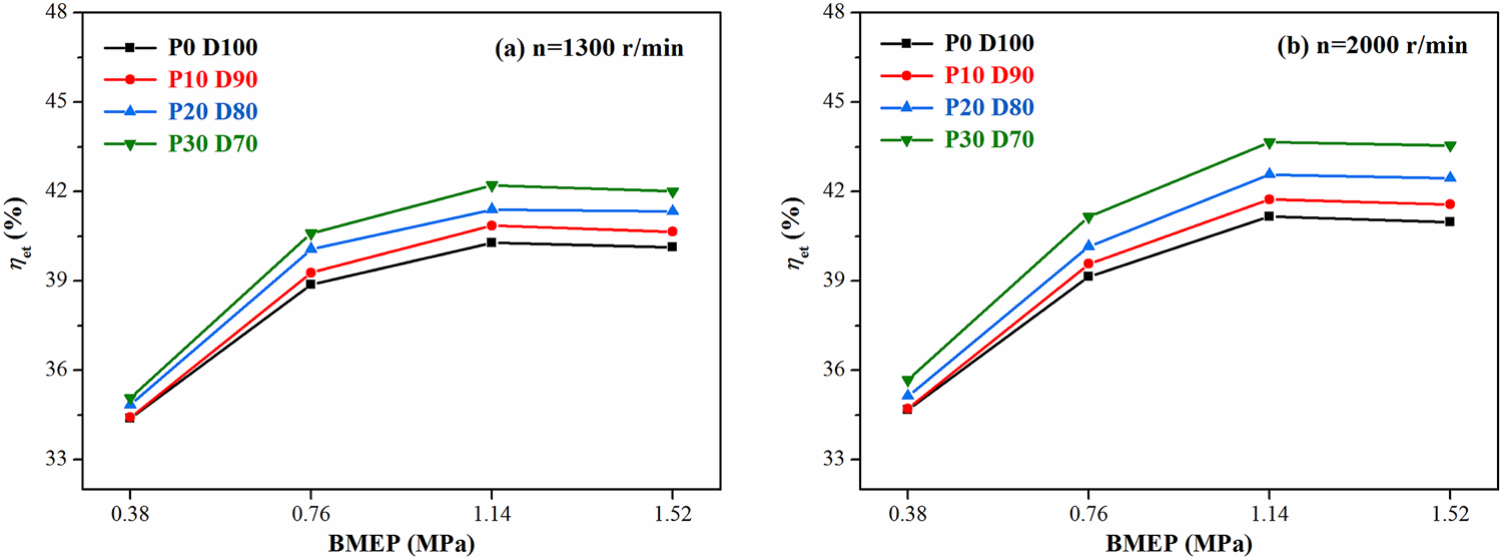
Effects of PODE_n_ blending ratio on effective thermal efficiency.

Figures 7 and 8 show the variations of the equivalent effective fuel consumption *b*_*eq*_ and the effective thermal efficiency *η*_*et*_ of the engine with different PODE_n_ blending ratios. Given the same load, with the increase in the PODE_n_ blending ratio, the equivalent effective fuel consumption gradually decreased, and the effective thermal efficiency increased significantly. With n = 2000 r·min^−1^ and BMEP = 1.52 MPa, compared with P0D100, the equivalent effective fuel consumptions of P10D90, P20D80, and P30D70 decreased by 2.20%, 3.26%, and 4.55%, respectively, with the effective thermal efficiencies increasing by 0.59%, 1.48%, and 2.57%, respectively. Due to the large cetane number of the PODE_n_, once the PODE_n_ were blended, the ignition delay period was shortened, and the combustion advanced with the heat release and the peak in-cylinder pressure in the main-injection phase being closer to the top dead center (Fig. 2), which was conducive to the improvement of the thermal efficiency. Furthermore, once the PODE_n_ were blended, the combustion duration shortened (Fig. 4), the heat released became more concentrated, and the low in-cylinder temperature (Fig. 3) reduced the heat transfer loss. Furthermore, with the increase in the PODE_n_ blending ratio, the oxygen content in the blended fuel increased, which reduced the area of oxygen-deficient combustion and improved the combustion reaction speed. In summary, blending PODE_n_ effectively improved the effective thermal efficiency of the engine and reduced the equivalent effective fuel consumption.

### Effects of blending PODE_n_ on emission characteristics of diesel engines

**Figure 9:**
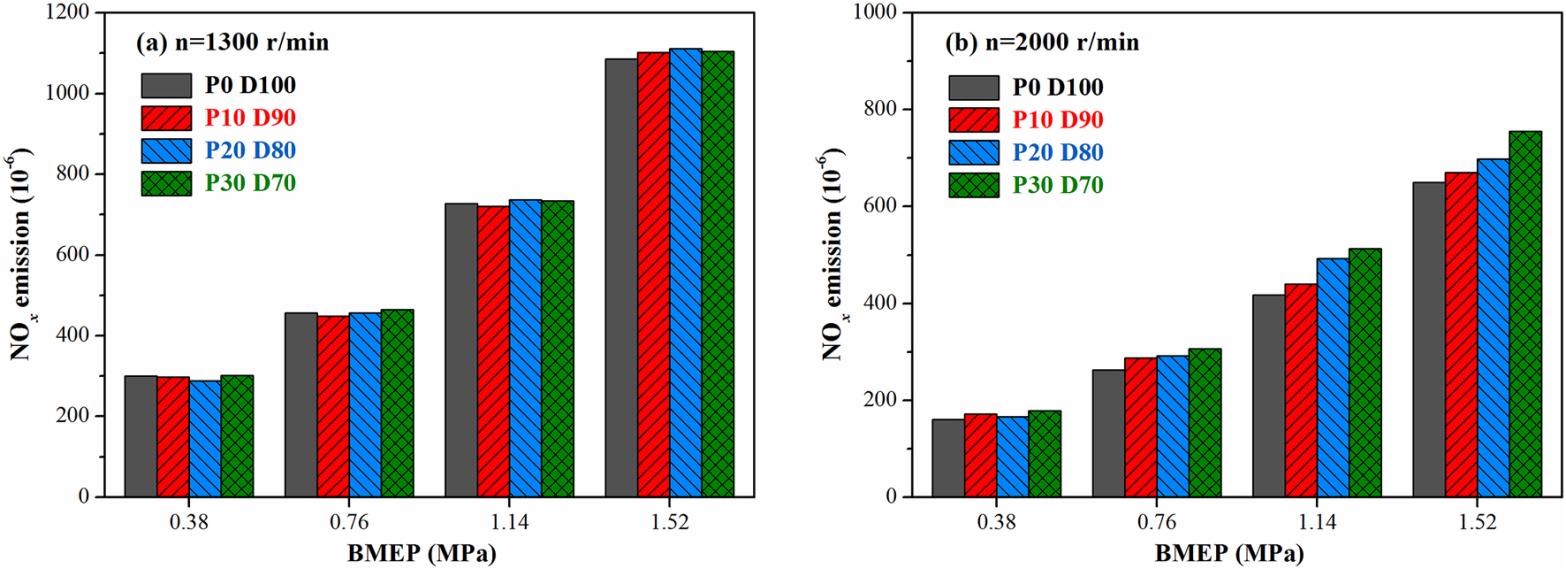
Effects of PODE_n_ blending ratio on NO_*x*_ emission.

High-temperature and oxygen-rich conditions with a long high-temperature duration are required for the formation of NO_*x*_^43^. Figure 9 shows the effect of the PODE_n_ blending on the engine emission of NO_*x*_ under different operating conditions. Under low-speed conditions (n = 1300 r·min^−1^), the in-cylinder temperature was low. As the PODE_n_ blending ratio increased, the in-cylinder temperature further declined (Fig. 3). Moreover, the shortened combustion duration shortened the high-temperature duration and suppressed the generation of NO_*x*_. However, the increased oxygen content in the high-temperature region increased the emission of NO_*x*_. Combining the two effects, blending PODE_n_ into the diesel fuel at different ratios made little difference under low-speed conditions. Under high-speed conditions (n = 2000 r·min^−1^), the in-cylinder temperature increased rapidly. Although blending PODE_n_ decreased the in-cylinder temperature, the in-cylinder temperature remained high (the peak temperature shown in Fig. 3 was about 2000 K). At that time, the NO_*x*_ was quite sensitive to the oxygen concentration in the cylinder during combustion, so the high oxygen content of the PODE_n_ rapidly increased the emission of NO_*x*_. In addition, the NO_*x*_ emission at a speed of 2,000 r·min^−1^ was generally lower than that at 1,300 r·min^−1^, which was likely because after the speed increased, the fuel reaction time was reduced, the high-temperature duration decreased, and the emission of NO_*x*_ decreased. At the same speed, as the load increased, the emission of NO_*x*_ increased due to the increase in the in-cylinder temperature.

**Figure 10:**
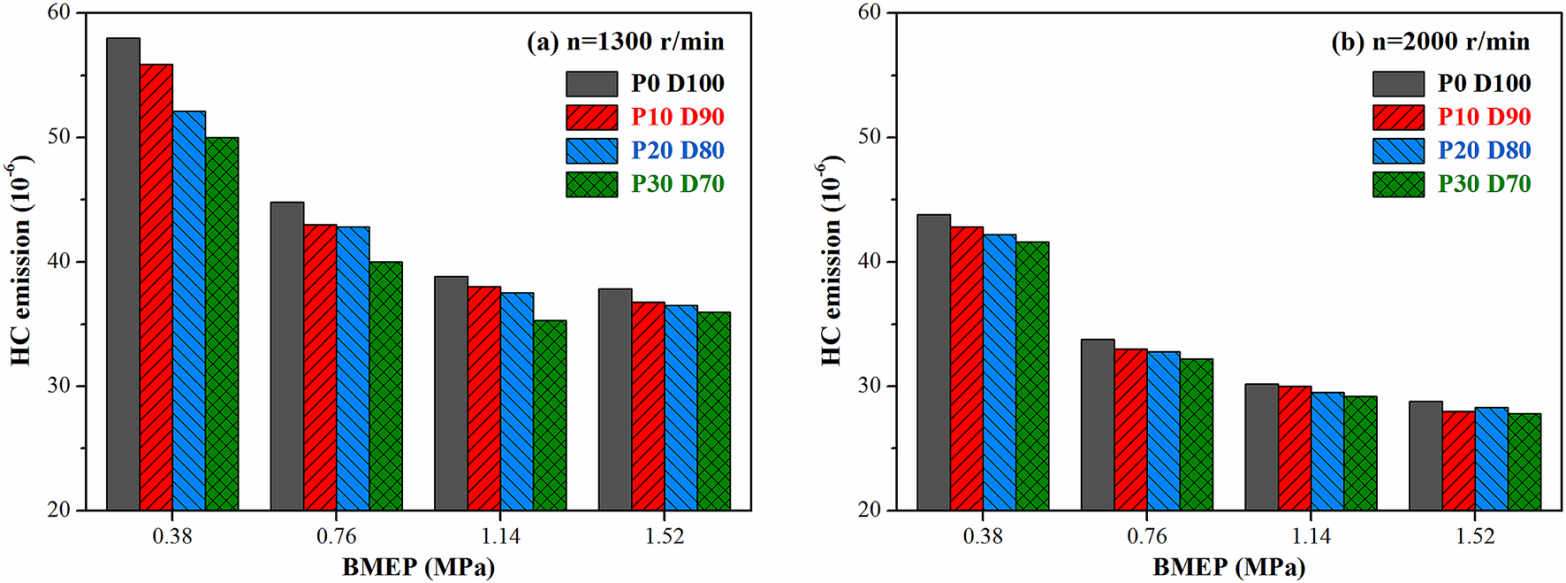
Effects of PODE_n_ blending ratio on HC emission.

The effect of PODE_n_ blending on the HC emission of the engine under different operating conditions is shown in Fig. 10. With n = 1,300 r·min^−1^, with the increase in the PODE_n_ blending ratio, the emission of HC gradually decreased. This was because the emissions of HC were mainly generated in the lean gas mixture and wall quenching layer around the injection fuel spray. The large cetane number of the PODE_n_ improved the ignition performance of the blended fuel and shortened the ignition delay period, thereby reducing the HC emission in the lean gas mixture and wall quenching layer. Moreover, due to the low boiling point and high oxygen content of the PODE_n_, the speed of mixing and combustion of oil and gas improved, which was beneficial for the oxidation of HC. With n = 2,000 r·min^−1^, the in-cylinder temperature was relatively high, and the emission of HC was low and decreased slightly with the increase in the PODE_n_ blending ratio. As the load increased, the emission of HC significantly decreased. This was because as the load increased, the in-cylinder temperature gradually increased, more HC was oxidized, and the emission of HC gradually decreased.

**Figure 11:**
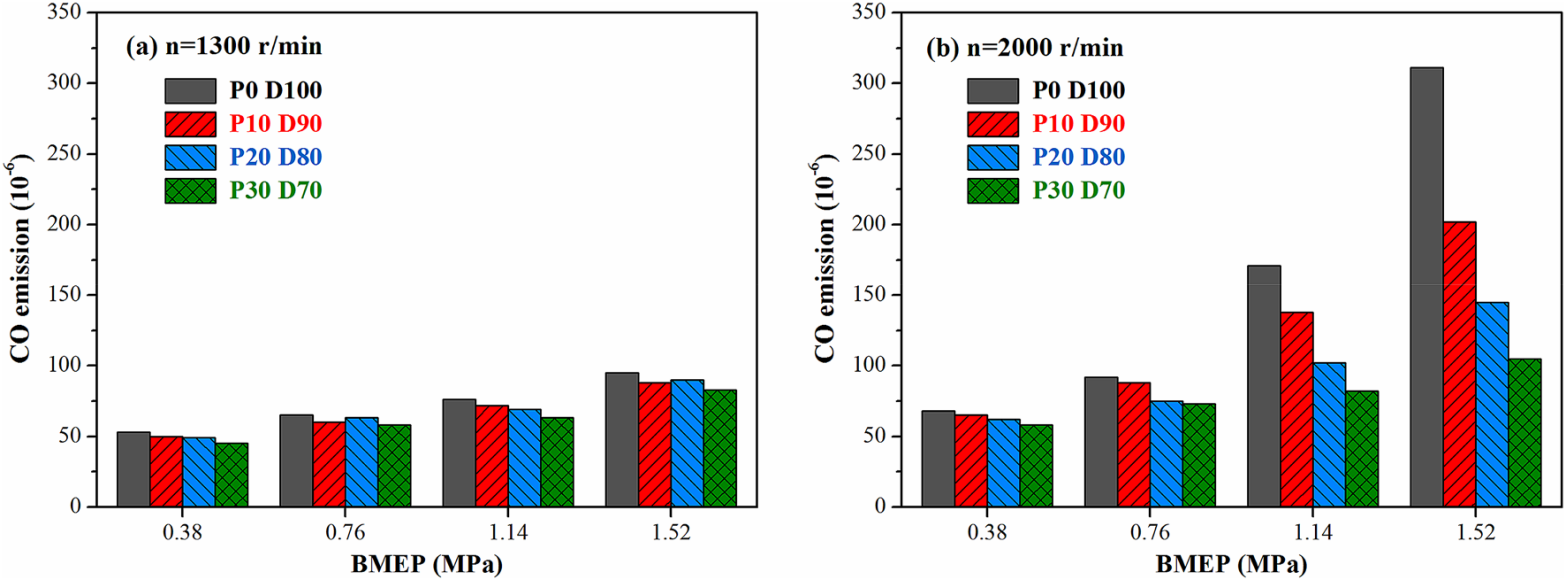
Effects of PODE_n_ blending ratio on CO emission.

The emission of CO was mainly generated in rich mixed gas areas or low-temperature areas where the excess air coefficient was less than 1. Figure 11 shows the effect of PODE_n_ blending on the CO emission of the engine under different operating conditions. At a speed of 1,300 r·min^−1^, the emission of CO was quite low when different fuels were used, and the emission changed slightly as the load increased. Since the excess air coefficient was large at low speeds, the emission of CO was comparatively low. At a speed of 2,000 r·min^−1^, the emission of CO declined slightly with the increase in the PODE_n_ blending ratio at low and medium loads, whereas at high loads, the emission of CO decreased significantly as the PODE_n_ blending ratio increased. With BMEP = 1.52 MPa, the CO emissions of P10D90, P20D80, and P30D70 were reduced by 35.0%, 53.4%, and 66.2%, respectively, compared with that of P0D100. At low and medium loads, the diesel engine basically worked with lean mixtures with an excess air coefficient greater than 1. There were fewer low-temperature and hypoxic areas in the cylinder, and the emission of CO was relatively low. Thus, the PODE_n_ had a comparatively small impact on the emission of CO. At high loads, there was a relatively rich mixture of local hypoxia in the cylinder, resulting in a rapid increase in the emission of CO. The high oxygen content of PODE_n_ increased the air–fuel ratio and excess air coefficient, improved the combustion conditions in the local hypoxic areas in the combustion chamber, and accelerated the oxidation rate of CO, which significantly decreased the emission of CO.

**Figure 12:**
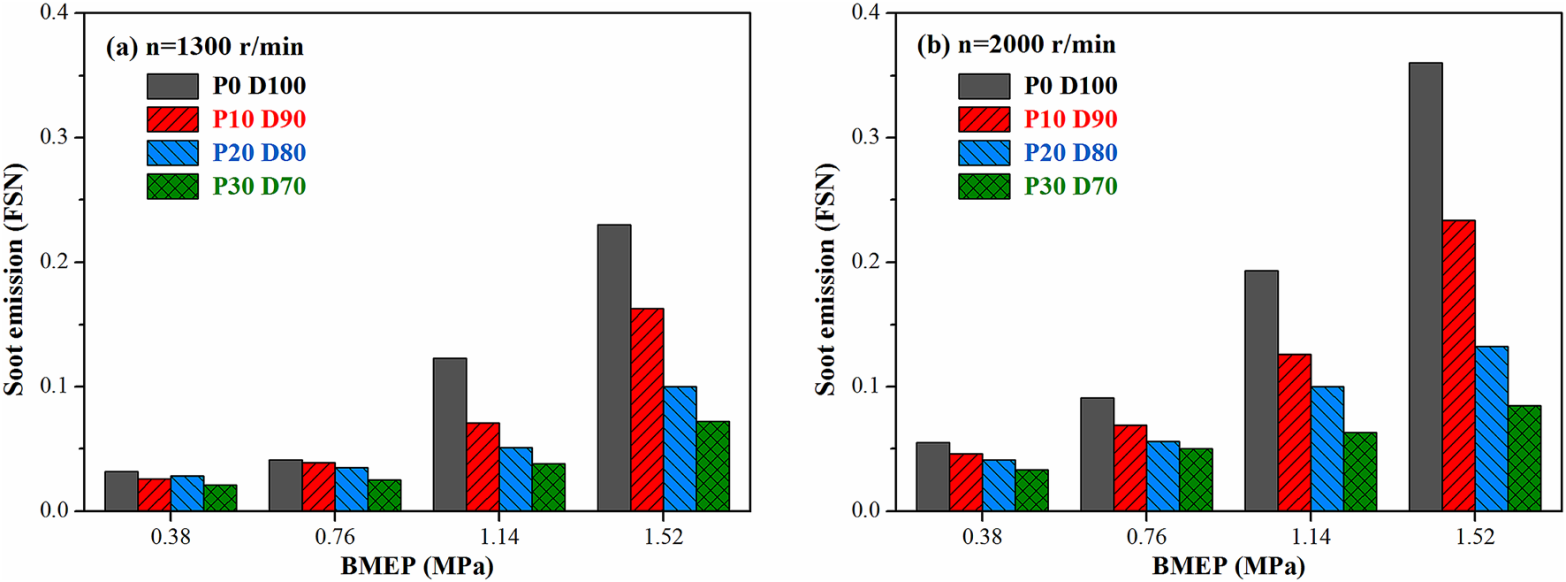
Effects of PODE_n_ blending ratio on soot emission.

Figure 12 shows the effect of PODE_n_ blending on the soot emission under different operating conditions. Blending PODE_n_ could effectively reduce the soot emission of diesel engines, and the reduction was more evident under large loads. With n = 2,000 r·min^−1^ and BMEP = 1.52 MPa, the filter smoke number (FSN) values of P10D90, P20D80, and P30D70 decreased by 35.1%, 62.8%, and 76.3% compared to that of P0D100. Unlike diesel, the PODE_n_ exhibited great volatility and a low viscosity and boiling point. Therefore, blending PODE_n_ contributed to the evaporation and atomization of the fuel, improved the homogeneity of the mixed gas, and effectively avoided fuel cracking caused by an uneven gas mixture under high-temperature and hypoxia conditions^44^. Furthermore, blending PODE_n_ increased the oxygen content in the blended fuel, which supplied the oxygen during the combustion process, thereby improving the combustion conditions in some areas during the diffusion combustion process of the diesel engine and promoting the post-oxidation process of the soot. In particular, the PODE_n_ molecules did not contain C–C bonds, which could significantly reduce the amounts of olefins and PAHs generated during the combustion reaction, thereby lowering the amount of soot. The above factors greatly improved the soot emission of the diesel engine after the blending of PODE_n_. Furthermore, under high speeds and loads, the soot emission decreased more substantially with the increase in the PODE_n_ blending ratio. With increases in the speed and the load, the combustion temperature in the cylinder increased, and the high-temperature hypoxic areas increased. Hence, due to the high oxygen content and excellent volatility, the PODE_n_ had a more evident effect on improving the combustion conditions when the gas mixture had an excessively high concentration.

## Conclusions

PODE_n_, with a high oxygen content and a large cetane number, are a promising additive for diesel. In this study, a series of tests were performed on a supercharged intercooled high-pressure common-rail diesel engine powered by diesel fuel blended with PODE_n_ at different ratios, i.e., 10%, 20%, and 30%, to explore the influences of PODE_n_ blending on the combustion and emission characteristics as well as the fuel economies of a heavy-duty diesel engine that met the China VI emission standards. The following conclusions were obtained:

1. Blending PODE_n_ significantly affected the combustion characteristics of the diesel engine. As the PODE_n_ blending ratio increased, the peak in-cylinder pressure and the average in-cylinder temperature gradually decreased, and the phase corresponding to the peak gradually shifted forward. The peak heat release rate in the pre-injection stage varied slightly, and the peak heat release rate in the main-injection stage gradually decreased, with the phase corresponding to the peak gradually moving forward.
2. As the PODE_n_ blending ratio increased, the start of the combustion gradually shifted forward, the duration of combustion was shortened by 0.7–2.8°CA, the heat release became more concentrated, the combustion heat release of the fuel was closer to the top dead center, and the maximum effective thermal efficiency could be improved by 2.57%. After blending with PODE_n_, the effective fuel consumption increased, but the equivalent effective fuel consumption decreased significantly, with the maximum reduction reaching 4.55%.
3. Blending PODE_n_ into diesel could effectively improve the emission characteristics of the diesel engines. Particularly under high-speed and high-load conditions, the PODE_n_, due to its high oxygen content and great volatility, improved the oxygen concentration in the cylinder and the uniformity of the combustible mixture, which effectively inhibited the generation of soot and reduced the emission of soot by as much as 76.3%. With the increase in the PODE_n_ blending ratio, the emission of NO_*x*_ increased slightly, but the emissions of HC and CO gradually decreased, with the highest reduction of the CO emissions reaching 66.2%.
